# Cerebrospinal Fluid (CSF) Proteomic Signature in Preclinical and Clinical Alzheimer’s disease (AD): Role of Adhesion Molecules

**DOI:** 10.21203/rs.3.rs-5404760/v1

**Published:** 2025-01-16

**Authors:** Reem Neal, Zhiyi Yang, Malik Obideen, Melissa Peterson, Amil Shah, Ihab Hajjar

**Affiliations:** 1 Department of Neurology, University of Texas Southwestern Medical Center, Dallas, Texas, USA 75390.; 2 Family Medicine and Osteopathic Manipulative Medicine, Texas College of Osteopathic Medicine, The University of North Texas Health Science Center; 3 Department of Medicine, University of Texas Southwestern Medical Center, Dallas, Texas, USA, 75390.

## Abstract

**Background:**

Although Amyloid-beta and Tau are the hallmarks of Alzheimer’s Disease (AD), other protein pathways such as endothelial dysfunction may be involved and may precede cognitive symptoms. Our objective was to characterize the cerebrospinal fluid (CSF) proteomic profiles focusing on cardiometabolic-related protein pathways in individuals on the AD spectrum.

**Methods:**

We performed CSF and plasma-targeted proteomics (276 proteins) from 354 participants of the Brain Stress Hypertension and Aging Program (BSHARP), of which 8% had preclinical AD, and 24% had MCI due to AD. We instituted a bioinformatic pipeline to generate data-driven protein modules, used “Hub” and “Critical” proteins within each module to describe protein signatures for each AD stage and then assessed their associations with clinical and biological AD traits. Finally, we completed pathway enrichment analysis to get insight into pathways that might be implicated in AD pathogenesis.

**Results:**

The 276 measured proteins clustered into five modules that were associated with CSF Amyloid-β42, Tau, and pTau. (all *p*-value <0.05). A CSF protein AD signature was characterized by elevated levels of CSF Hepatocyte Growth Factor (*HGF*), Intercellular and Vascular Cell Adhesion Molecule 1 (ICAM-1, *VCAM-1*), Neuropilin 1 and 2 (*NRP-1*, *NRP-2*), Scavenger Receptor Class B Member 2(*SCARB2*), Plasminogen Activator, Urokinase (*PLAU*). *(all <0.05)* We also found a significant difference in the CSF/Plasma ratio for the proteins associated with Cognitive Status and the Tau/Aβ42 ratio (TAR) in the CSF. Pathway enrichment analysis revealed that cell adhesion and endothelial dysfunction (all *p*-value <0.05) were key mechanisms involved in AD pathogenesis, especially in the preclinical stage.

**Conclusion:**

Our results suggest a proteomic signature in the CSF of individuals with preclinical AD that is driven by adhesion molecules and might be implicated in the pathogenesis of AD. Future studies investigating these pathways may provide insights into novel AD biomarkers and therapeutic targets.

## 1. Introduction:

Alzheimer’s disease (AD) is the leading cause of dementia.^1,2^ It is a proteinopathy characterized by a progressive decline in cognitive function and the accumulation of the Amyloid βeta and Tau in the brain (AD hallmark).^3^ The cerebrospinal fluid (CSF) proteins amyloid βeta1–42 (*Aβ42*), *Tau*, phosphorylated tau (*pTau*), and the Tau/Aβ42 ratio (TAR) are considered accurate measures that reflect those brain pathologies.^4^ However, despite the significant efforts put into developing treatments that target the accumulation of Aβ and Tau, the treatment of AD remains a significant challenge. Therefore, there is a crucial need for understanding the molecular mechanisms associated with the accumulation of Aβ and Tau-related pathologies to develop alternative therapies.

Previous studies have supported the notion that AD is multifactorial and involves a complex set of molecular mechanisms.^5,6^ The suggested mechanisms include pathways that are related to metabolism, neuroinflammation, and cardiometabolic and endothelial dysfunction.^7,8^ Moreover, it has been suggested that AD progression is likely more rapid in patients with prodromal AD who have higher cardiovascular risk.^9^ Prior studies have also suggested links between cardiometabolic factors and tau pathology in patients with preclinical AD.^10^ This highlights the need for expanding our research to include pathways beyond those centered around Aβ and Tau pathologies during its early phases. Identifying novel AD biomarkers upstream or downstream of changes in CSF AD biomarkers during the preclinical and prodromal stages of AD might offer insight into the mechanisms underlying AD onset or progression. Furthermore, it would be a major step towards developing novel therapeutic options for AD patients.^7^

Advances in proteomic studies and methods of protein detection have offered new opportunities to gain insights into the molecular pathways contributing to a disease’s pathophysiology.^11–15^ Moreover, studies applying advanced bioinformatics such as network-based approaches have demonstrated their ability to help detect novel proteins and identify the mechanisms involved in alternative disease pathways.^2,16–18^ In this study, we aimed to characterize the cerebrospinal fluid (CSF) proteomic profiles of individuals on the AD spectrum.

## 2. Methods

### 2.1 Participants:

We recruited 375 participants to The Brain Stress Hypertension and Aging program (B-SHARP) in Atlanta, GA. Participants were identified either through a referral from the Emory Goizueta Alzheimer’s Disease Research Center or through strategic community partnerships with grass-roots health education organizations, health fairs, advertisements, and mail-out announcements. An appropriate study informant, defined as an individual who has regular contact with the participant for at least once a week (in person or by telephone), was also identified. All participants attended a screening visit, during which they signed a written informed consent and underwent cognitive testing. Demographics (age, sex, race, education), anthropometrics (weight and height), medical diagnosis, and income levels were collected at baseline by interview. A study physician performed clinical evaluation and lumbar punctures (LP).

We used data from participants in the Health and Aging Brain Study: Health Disparities (HABS-HD) with MCI and AD was utilized for validation of our results in a different population. In short, the HABS-HD study is a large multi-ethnic cohort at the University of North Texas Health Science Center, Fort Worth, Texas, that studies factors influencing the health disparities and mechanisms involved in MCI and AD.^19–21^ Details of the study have been previously described. ^22^

### 2.2 Cognitive assessments:

MCI was determined by cognitive assessments and a consensus conference including physicians and neuropsychologists. MCI was defined as having the following: Montreal Cognitive Assessment (MoCA) score of less than 26, subjective memory concerns, Clinical Dementia Rating (CDR) sum of boxes score of 0.5 and memory box score of 0.5, education-adjusted cutoff score on the Logical Memory task (delayed paragraph A only) of the Wechsler Memory Scale–Revised (Alzheimer Disease Neuroimaging Initiative) of less than 11 for 16 or more years of education, less than 9 for 8 to 15 years of education, and less than 6 for 7 or fewer years of education (the maximum score is 25), and preserved instrumental activities of daily living (Functional Activities Questionnaire) score of 7 or less.

### 2.3 Lumbar punctures (LP):

LP procedures were performed following a fast of no less than 6 hours using 24G Sprotte atraumatic spinal needles. Samples were collected in sterile polypropylene tubes, separated into 0.5cc aliquots, and stored at −80 °C.

### 2.4 AD Biomarkers and targeted protein Measurement:

Aβ_42_, Tau, and pTau assays were performed following a single freeze-thaw cycle on a Roche Cobas e601 analyzer using the Elecsys Gen1 platform^23^ following manufacturer’s protocols. Plasma AD biomarkers were measured using the Simoa Platform Version 2 Advantage Kit (Quanterix Corp) and were used in a fully automated 2-step sandwich immunoassay as described previously^24^.

We conducted CSF proteomics using three 92- Olink panels. The panels measured markers of neuronal injury, cardiometabolic and inflammation. The proximity extension assay (PEA) technology was used to quantify proteins in 9 plates of plasma samples and 6 plates of CSF samples. Briefly the Olink proximity extension assay (PEA) includes the use of sensitive immunoassays that utilize two probe oligonucleotides (specific to the proteins of interest) and qPCR.^25^ Each sample has four internal controls for quality control (QC) of the sample and the assay. The output data includes data from all samples and a “QC Warning” column for samples that do not pass QC. Protein data is presented as Normalized Protein Expression (NPX), an arbitrary unit on the Log2 scale that reflects protein concentration. Although NPX does not reflect the exact protein concentrations, a high NPX value corresponds to a high protein concentration. The data file with the NPX values includes the data of all samples with data of samples that did not pass QC being in red text. The data for measurements below the limit of detection (LOD) have a pink background.

### 2.5 Independent corroboration of the association of adhesion molecules with AD Biomarkers:

We elected to use the available HABS-HD dataset because it includes concurrent blood based measurements of adhesion molecules and AD biomarkers as a method to corroborate our findings.^22^ The HABS-HD data set included measurements of plasma AD biomarkers such *Aβ42, Tau, pTau* and adhesion molecules such as *VCAM* and *ICAM* measured by the ultra-sensitive Simoa (single molecule array) technology platform (Quanterix.com).^22^

### 2.6 Statistical analysis:

Statistical analysis was performed with R (version 4.2.2). The statistical significance was defined as a 2-sided P < 0.05. We used one-way ANOVA or χ2 test to compare study participants’ characteristics which are presented as means (SDs) or numbers (percentages).

Co-expression networks were constructed using the MONET K1 algorithms.^26^ The Kernel clustering optimization algorithm (K1) is based on the “Diffusion State Distance” (DSD).^27^ The DSD metric defines a pairwise distance matrix between all nodes, and then applies a spectral clustering algorithm. It also uses standard graph techniques to identify dense bipartite sub-graphs. Then both results are merged into a single set of non-overlapping clusters. The proteins in our study clustered into 5 modules ranging from 80 proteins (M1) to 14 proteins (M5).

The Hub proteins were defined as the top 10% of proteins associated with clinical traits and have the most connectivity to other proteins in each module in addition to having met the absolute value of the protein-Module Membership >0.80 (High correlation of a protein expression profile with the module eigengene of a given module) and protein-Trait Significance >0.20 (biological significance of the protein with an external microarray sample trait). After identifying Hub proteins, the STRING, version 12.0^28^ database was used to construct a protein-protein interaction (PPI) network and Cytoscape^29^ was applied to visualize the PPI network. Proteins with high degrees of intramodular connectivity in the PPI networks, were defined as critical proteins.^30^

Pathway enrichment analysis was performed to understand the biological significance of the hub proteins identified. Gene Ontology (GO) pathway analysis weas performed using Enrichr a web-based data-analysis tool.^31,32^ The top enriched biological and molecular pathways were then displayed in a bar graph based on the *P*-value (-log10). The term at the top has the most significant overlap with the input query gene set.

## 3. Results:

### 3.1 Sample characteristics:

Our study included 354 participants recruited from the B-SHARP cohort with available CSF samples. Participants had a mean age of 65 and gender and race distribution consisted of 223 women (63%), 131 men (37.01%), 144 African American participants (40.7%) and 210 White patients (59.3%). The sample included 104 (29.4%) participants with MCI and negative AD biomarkers. There were 116 (32.8%) participants that had positive AD biomarkers of which 29 participants had preclinical AD and 87 participants that had prodromal AD. The key characteristics of the study participants are provided in Table 1.

### 3.2 Module Design & Identification of Hub and Critical Proteins:

**Figure.2:**
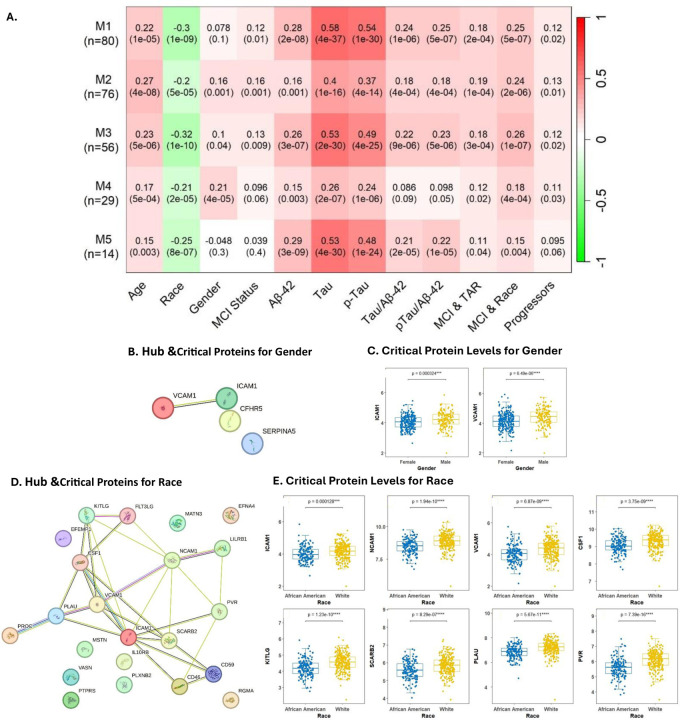
Association of protein modules with AD clinical phenotypes: **A.** Heat map showing the association between protein modules and both clinical and biological AD phenotypes. Each cell contains the corresponding correlation and p-value, each row corresponds to a module, and each column corresponds to a feature. **B.** A Protein-Protein interaction (PPI) network of the Hub proteins associated with gender. **C.** Box plots of expression levels of Critical Protein associated with gender. **D.** A Protein-Protein interaction (PPI) network of the Hub proteins associated with race. **E.** Box plots comparing the expression of Critical Proteins associated with race.

Targeted proteomics were performed on CSF samples to analyze 276 proteins. We then applied a bioinformatic pipeline to generate data-driven protein modules which resulted in the proteins being clustered into 5 modules that varied in their degree of association with age, race, CSF AD biomarkers (Aβ42, Tau, and pTau) and clinical phenotypes such as disease progression, cognitive function, and the Aβ42/Tau Ratio (TAR). The modules ranged in size from 80 proteins (M1) to 14 proteins (M5). (Figure.2A)

Next, we identified Hub proteins and critical proteins for each clinical phenotype and CSF AD biomarker. Proteins with the highest connectivity and correlation with other proteins and a selected AD-related clinical trait were selected as Hub proteins. We found 22 Hub proteins for Race, 4 Hub proteins for gender, 1 Hub protein for having MCI, 12 proteins for Aβ42, 23 proteins for Tau, 23 proteins for pTau, 21 proteins for the Tau/Ab42 ratio, 13 proteins for both cognitive function and TAR status, and 13 proteins Hub proteins for disease progression. To identify critical proteins, we used the STRING database to construct protein-protein interaction (PPI) networks with the hub proteins and selected the proteins with the highest intramodular connectivity in the PPI network. (Table.2 & Figures 2B & 2D) (Fig S.1)

### 3.3. Association of Hub & Critical proteins with Demographic & Clinical AD-related phenotypes:

Sex and Race have been shown to play a significant role in the pathophysiology of many diseases. Therefore, we explored whether there were proteomic differences associated with sex and Race. Our analysis revealed that the levels of the 8 critical proteins identified for race differed significantly between African American participants and white participants (all *P<0.05*). (Figure.2D-E) For sex, we found that the levels of only 2 of the critical proteins (*ICAM1, VCAM1*) significantly differed between female and male participants (both *P<0.05*). (Figure.2B-C).

We also explored the proteomic difference based on clinical traits such as having an MCI diagnosis and disease progression. We identified only one hub protein for having an MCI diagnosis (*TNFRSF12A*) which had significantly lower expression in participants without an MCI diagnosis (*P=0.0004*). For disease progression although we had identified 4 critical proteins, only the levels of (*CCL3)* differed between progressors and non-progressors(*P=0.05*). (Fig S.2). Next, we wanted to identify proteomic biomarkers that might be associated with AD pathology during the preclinical and prodromal stage of AD.

### 3.3 Association of Hub & Critical proteins with AD Biomarkers and cognitive function:

**Figure.3:**
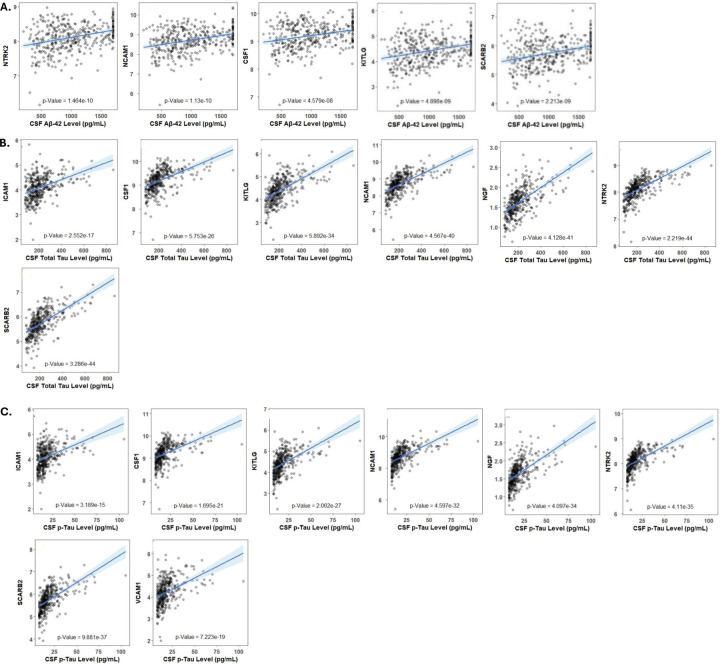
Association of protein modules with AD Biological phenotypes (CSF AD Biomarkers): The correlation between critical proteins and levels of CSF AD biomarkers: **A.** Aβ42**, B.** Tau, and **C.** pTau.

As CSF AD biomarkers reflect AD pathology in the brain, we assessed the correlation between the levels of CSF AD biomarkers (Aβ42, Tau, pTau), the Tau/Aβ42 ratio (TAR) and the critical proteins we identified. We found significant correlations between all CSF AD biomarkers and the Tau/Aβ42 with the critical proteins (Figure.3A-C & Fig S.3) In our blood samples we observed significant correlations between blood-based AD biomarkers and adhesion molecules. We found that plasma Aβ42 had significant correlations with both *ICAM* and *VCAM*. Likewise, plasma Tau also had significant correlations with both *ICAM* and *VCAM*. (Table S.1).

**Figure 4:**
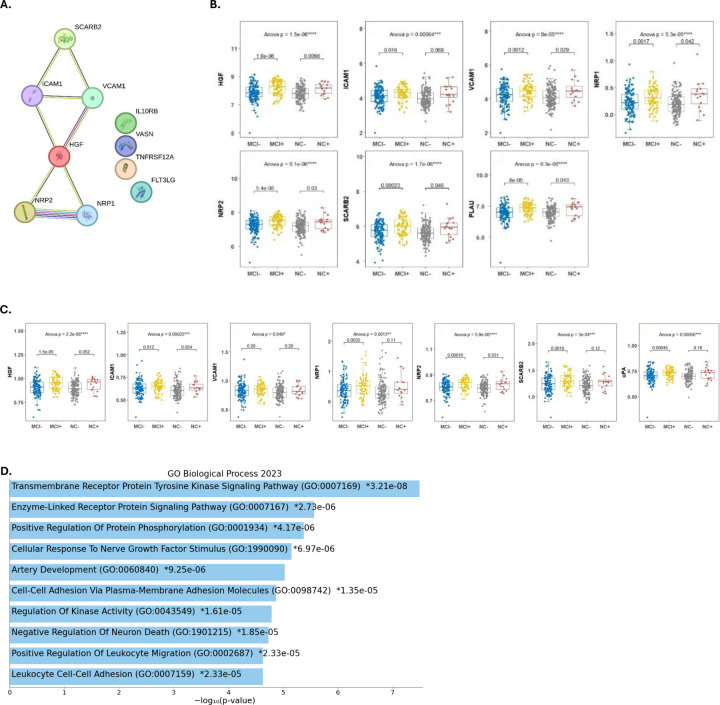
AD CSF Proteomic signature in the preclinical and prodromal stages: **A.** Hub and Critical proteins PPI network for cognitive & TAR status **B.** Box Plots comparison of the CSF levels of Critical Proteins (CSF) associated with Cognitive function & TAR status between participants with normal cognition (NC-), preclinical AD(NC+), prodromal AD(MCI+), and participants with non-AD MCI (MCI-) **C.** Box Plots comparison of the CSF/Plasma Ratio of Critical Proteins associated with Cognitive function & TAR status between the 4 groups. **D.** Pathway Enrichment Analysis: Bar chart of top enriched terms from the GO_Biological_Process_2023 gene set library.

To further understand the contribution of these proteins to the pathophysiology of AD, we identified Hub and Critical proteins associated with both cognitive function and TAR status. (Figure.4A) We then conducted a Pathway enrichment analysis of those proteins to identify the possible underlying biological mechanisms that might be impacted in AD. This revealed that pathways related to cell adhesion and vascular function (all *P*<0.05) might play an important role in AD. (Figure.4D)

### 3.4 Proteomic differences associated with AD stages (4 group comparison):

Our subsequent analysis aimed to further explore the previous finding. Therefore, we compared the proteomic profiles of participants with normal cognition (NC-), preclinical AD (NC+), prodromal AD (AD-MCI), and non-AD MCI (NAD-MCI) using the critical proteins associated with both cognitive function and TAR status. We found that six of the critical proteins identified for both cognitive function and TAR status (*HGF, ICAM1, VCAM1, NRP1, NRP2, SCARB2, PLAU*) were differentially expressed between the four groups (all had a of *P<0.05*). (Figure.4B)

As for blood-based AD biomarkers we found that only *VCAM* significantly differed between the participants with AD MCI and Non-AD MCI (*P*-value <0.009). (Fig.S4) However, when we compared the CSF/Plasma ratio among the four groups, we found (*HGF, ICAM1, VCAM1, NRP1, NRP2, SCARB2, PLAU*) were all significantly differentially expressed. (Figure.4C)

### 3.5 Association of Adhesion Molecules with AD Biomarkers in HABS-HD dataset:

Given the availability of the HABS-HD data set we wanted to see if we could replicate or find similar observations in a different cohort. As observed in our BSHARP dataset, we found significant associations in the HABS- HD dataset between *VCAM* and all 3 AD biomarkers Aβ42, Tau, and pTau (all *P*<0.0001). However, *ICAM* and *NCAM* only significantly correlated with Tau and Aβ42 respectively (both *P*<0.05). (Table S.2) This suggests that the critical proteins we identified, specifically adhesion molecules, might play an important role in the pathophysiology of AD especially during its preclinical and prodromal stages.

## 4 Discussion:

Alzheimer’s Disease (AD) is the most common cause of dementia^1^ and currently represents an irreversible disease.^2^ However, recent studies have suggested that the early stages of AD when patients have mild cognitive impairment (MCI), a precursor to dementia, represents an opportunity to intervene and attempt to halt the progression of AD. Hence, in this study we aimed to identify novel biomarkers that are associated with changes in the levels of AD biomarkers during the early stages of AD that might be contributing to the development and progression of AD.

Our study used targeted proteomics of CSF and plasma samples from 354 participants to identify Hub and Critical proteins that are significantly associated with clinical AD related phenotypes and CSF AD biomarker (Aβ42, Tau, pTau) as markers of AD pathology. The phenotypes included sex, race, disease progression, MCI diagnosis, and both TAR & cognitive status. The critical proteins we identified were also significantly correlated with blood-based AD biomarkers. Furthermore, we observed similar results in a different population. Pathway enrichment analysis of these proteins revealed that they were involved in pathways related to adhesion, inflammation, and vascular function -all known contributors to the pathophysiology of AD. Our findings also revealed a significant difference in levels of CSF critical proteins between TAR positive and TAR negative participants. The critical proteins included Hepatocyte growth factor (*HGF*), Intercellular cell adhesion molecule 1 (*ICAM-1*), Vascular cell adhesion molecule 1 (*VCAM-1*), Neuropilin 1 (*NRP-1*), Neuropilin 2 (*NRP-2*), Scavenger Receptor Class B Member 2 (*SCARB2*), and Plasminogen Activator, Urokinase (*PLAU*). This suggests that there is a proteomic signature in the CSF of individuals with AD during the preclinical and prodromal stage of AD that is driven by adhesion molecules. Therefore, adhesion molecules and proteins involved in endothelial dysfunction might have a significant role in the development and progression of AD.

Our findings align with previous studies that have reported associations between proteins such as *HGF, ICAM-1,* and *VCAM1* with CSF AD biomarkers, and faster decline in cognitive function.^33–37^ Furthermore, previous studies have revealed the contribution of these proteins to AD such as demonstrating that induction of endothelial *ICAM-1 leads to* decreased amyloid-β degradation.^38^ Similarly, they have shown that disrupting the interaction between *ApoEs* and *VCAM1* causes decreased microglial clearance of Aβ.^39^ These findings further support our conclusion of a significant role for adhesion molecules in AD.

Furthermore, the adhesion Molecules *ICAM-1* and *VCAM1* which are released from the endothelial membrane, are considered markers of vascular dysfunction -a major contributor to endothelial dysfunction.^40^ A previous study by Pillai et al. found CSF levels of *VCAM-1* and *ICAM1* to be reflective of endothelial damage in the blood brain barrier (BBB).^41^ Therefore, our findings also support previous studies that have suggested a role for vascular dysfunction in the development and progression of AD.^42^ Especially given our observation of a significant difference in the CSF/Plasma ratio of these proteins between TAR positive, and TAR negative participants which suggests a critical role for the involvement of the blood brain barrier (BBB) in AD.^41^

The association of AD with dysfunction of the BBB is well documented with evidence from previous studies demonstrating the role for *HGF, ICAM-1,* and *VCAM-1* in the permeability and breakdown of the blood brain barrier (BBB) through their role in vascular and endothelial function.^34*–*37,41,43,44^ Additionally, it has been shown that BBB dysfunction is associated with cognitive decline during the early stages of AD.^45^ This has been suggested to be attributed to the increased permeability of the BBB which results in increased entry of toxins into the central nervous system (CNS) causing neuroinflammation.^40^ This could possibly explain the variation in cognitive changes that are observed during the preclinical and prodromal stages of AD despite both stages being associated with the accumulation of AD pathologies in the brain.

Furthermore, our finding of *NRP1* among the critical proteins supports the role for both inflammation, and endothelial dysfunction in the pathophysiology of AD. *NRP1* has previously been identified as a mediator of the inflammatory response of endothelial cells in the BBB.^46^ This could explain the increased levels of adhesion molecules in TAR positive participants we observed by the overproduction of inflammatory cytokines which are known to activate the endothelium and induce the production of adhesion molecules. Therefore, our findings suggests that during the early stages of AD, adhesion molecules, especially those related to vascular function might play a significant role in the disruption of the BBB by mediating the neuroinflammation and endothelial dysfunction that are involved in the early pathophysiology of AD leading to further accumulation of AD pathologies in the brain. This aligns with the previously described synergistic effect of inflammation, and vascular dysfunction on the development and progression of AD.^47^

In summary our study has identified novel biomarkers that are associated with the preclinical and prodromal stage of AD. It contributes to our growing knowledge of the mechanisms involved in the early stages of AD. It also offers insight into novel protein markers that might be used for diagnostic purposes or as drug targets for novel therapeutics for AD. However, it is important that we acknowledge the limitations of our study such as its’ cross-sectional nature which means that the protein measurements were conducted only at a single point in time and cannot establish causality. Therefore, further investigation of these associations and their impact on disease trajectory in longitudinal studies is required. Despite these limitations our findings remain significant and future studies should further explore the molecular contribution of adhesion molecules in both the central nervous system (CNS) & the periphery and their involvement in the underlying biological mechanisms affected in AD.

## 5 Conclusions

Our findings revealed that adhesion, inflammation, and endothelial-related pathways are highly associated with AD phenotypes, especially during the preclinical stage of AD. They also suggest that there is a proteomic signature in the CSF of individuals with preclinical AD that is driven by adhesion molecules contributing to the pathogenesis of AD. Future studies investigating these contributions might provide insight into the molecular mechanisms underlying AD.

## Supplementary Material

Supplement 1

## Figures and Tables

**Figure.1:**
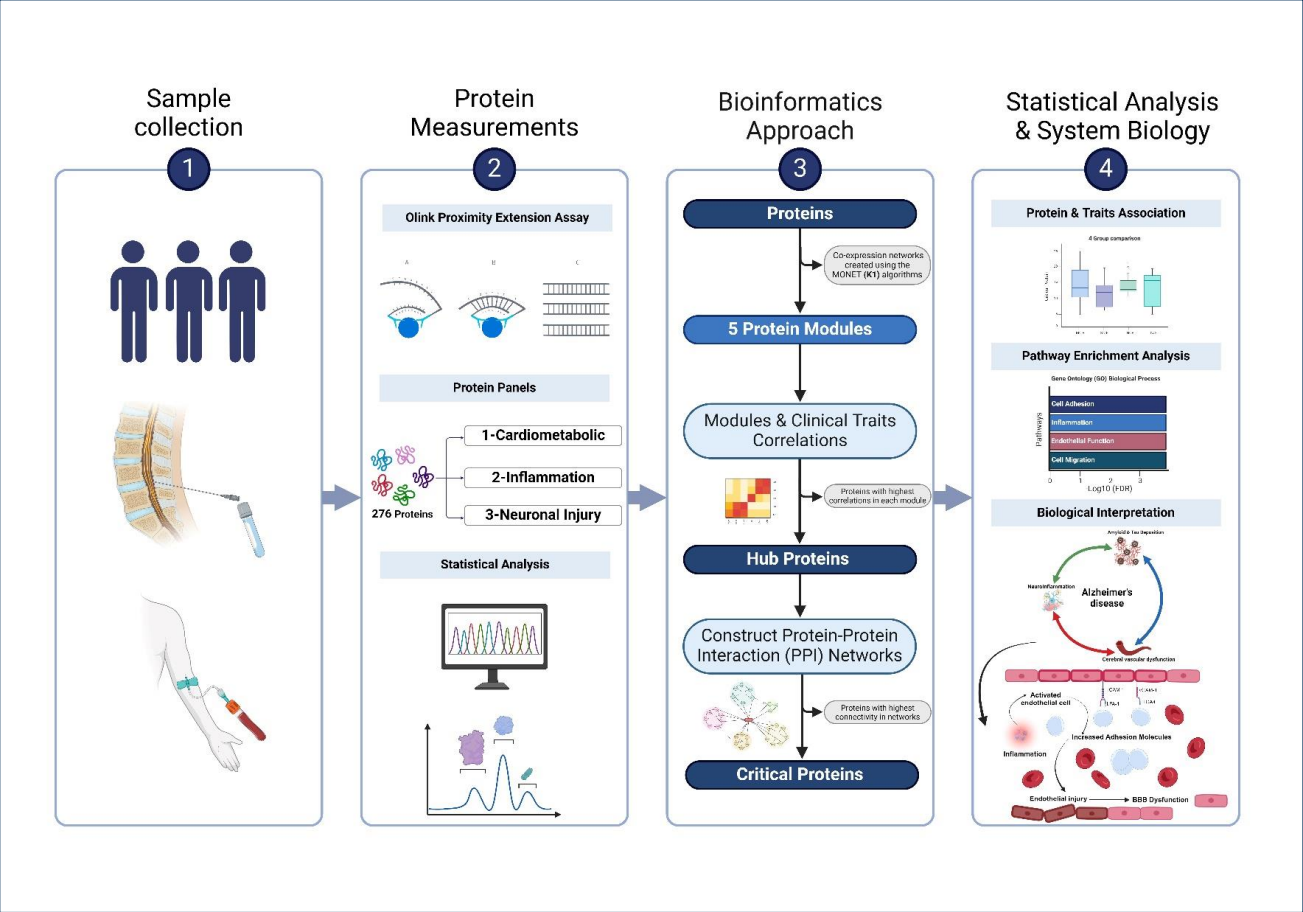
Schematic outline of study design: **A.** Blood and CSF sample collection from 354 Participants. **B.** Proteomic analyses of 3 Olink panels that include 92 markers each using Olink proximity extension immunoassays. **C.** Bioinformatic pipeline. **D.** Comparison of the expression of critical protein between participants with normal cognition (NC-), preclinical AD(NC+), prodromal AD(MCI+), and non-AD MCI (MCI-) followed by a Pathway enrichment analysis and a schematic diagram illustrating the contribution of neuroinflammation and vascular dysfunction to AD pathophysiology.

**Table 1.** Basic Demographic, Clinical, Biomarkers, & Imaging Comparisons between 4 Groups:*

Characteristic		MCI-	MCI+	NC-	NC+	*P-*Value^†^
***N***		104	87	134	29	
Age, mean (SD), years		64.17 (8.13)	70.91 (7.22)	62.73 (6.82)	65.55 (7.68)	<0.001
**Gender**	Female (%)	61 (58.7)	54 (62.1)	89 (66.4)	19 (65.5)	0.654
	Male (%)	43 (41.3)	33 (37.9)	45 (33.6)	10 (34.5)	
**Race**	AA (%)	68 (65.4)	14 (16.1)	56 (41.8)	6 (20.7)	<0.001
	White (%)	36 (34.6)	73 (83.9)	78 (58.2)	23 (79.3)	
Years of Education, mean (SD)		15.00 (2.65)	15.68 (2.72)	15.99 (2.53)	16.79 (2.62)	0.003
BMI, mean (SD), Kg/m^2^		29.88 (6.22)	25.27 (4.60)	28.77 (6.24)	27.50 (5.81)	<0.001
GFR, mean (SD)		81.08 (20.11)	74.80 (14.06)	82.65 (16.50)	79.72 (15.96)	0.010
Systolic BP, mean (SD), mmHg		129.75 (19.08)	126.65 (16.63)	126.02 (18.98)	122.78 (16.28)	0.231
**Clinical Diagnosis**						
Hypertension, N (%)		63 (60.6)	20 (23.0)	81 (61.4)	14 (48.3)	<.001
Diabetes Mellitus, N (%)		23 (22.1)	7 (8.0)	13 (9.8)	4 (13.8)	0.015
**Cognitive Status**						
HVLT-R Trial 4, mean (SD)		7.54 (2.69)	4.34 (3.78)	9.56 (2.06)	9.83 (1.77)	<0.001
TMT Part A, mean (SD), s		40.03 (16.31)	45.91 (23.07)	35.39 (15.11)	33.86 (11.24)	<0.001
TMT Part B, mean (SD), s		137.32 (83.82)	154.46 (84.21)	87.70 (44.11)	92.83 (63.28)	<0.001
TMT Part B - A, mean (SD), s		97.29 (76.19)	108.55 (81.73)	52.31 (37.66)	58.97 (59.42)	<0.001
MoCA score, mean (SD)		21.90 (3.11)	20.67 (3.41)	26.19 (2.67)	26.48 (2.18)	<0.001
**Neuroimaging Measures**						
Hippocampal Volume, mean (SD), mm^3^		7373.56 (936.51)	6380.46 (1000.98)	7567.25 (852.16)	7443.55 (1039.07)	<0.001
Cortical Thickness, mean (SD), mm		2.34 (0.10)	2.28 (0.09)	2.34 (0.09)	2.35 (0.07)	<0.001
**Biomarkers**						
Aβ42 (CSF), mean (SD), pg/ml		1156.36 (385.65)	589.76 (193.86)	1140.60 (377.81)	686.26 (215.64)	<0.001
Tau (CSF), mean (SD), pg/ml		169.80 (58.36)	328.10 (148.95)	168.39 (56.60)	256.42 (85.52)	<0.001
p-Tau (CSF), mean (SD), pg/ml		14.20 (4.90)	31.46 (16.96)	13.75 (4.41)	21.16 (8.06)	<0.001
Tau/ Aβ42 (CSF) Ratio, mean (SD)		0.15 (0.04)	0.61 (0.39)	0.16 (0.05)	0.39 (0.13)	<0.001
**Olink Proteins**						
ICAM1 (CSF), mean (SD)		4.09 (0.44)	4.24 (0.42)	3.98 (0.43)	4.19 (0.50)	<0.001
NCAM1 (CSF), mean (SD)		8.63 (0.56)	8.88 (0.59)	8.60 (0.51)	8.85 (0.58)	0.001
VCAM1 (CSF), mean (SD)		4.18 (0.53)	4.43 (0.55)	4.08 (0.52)	4.39 (0.56)	<0.001

* **Abbreviations: -NC**: Normal Cognition, **MCI**: Mild Cognitive Impairment, **SD**: standard deviation, **N**: Sample size, **AA**: African American, **BMI**: Body Mass Index, **GFR**: Glomerular Filtration Rate**, BP**: Blood Pressure, **HVLT -R**: Hopkins Verbal Learning Test Revised, **TMT**: Trail Making Test, **MOCA**: Montreal Cognitive Assessment, **Aβ42**: Amyloid-β42, **Tau**: Tau protein, **pTau**: Phosphorylated Tau protein, **CSF**: Cerebrospinal Fluid.

† *P*-values obtained through a pooled analysis using t-test for continuous data, and chi-square for discrete data.

**Table.2:** Hub and Critical proteins:*

Trait	Hub proteins	Critical Proteins
**Age**	*HGF, EFNA4, NRP2, VASN, TGFBR3, PLAU, RGMA, PLXNB2, TNFRSF12A, FLT3LG, IL10RB, KITLG, VCAM1, ICAM1, CSF1, SPOCK1, PVR, SCARB2, LILRB1*	*CSF1, ICAM1, VCAM1, HGF, KITLG, PLAU, PVR, SCARB2*
**Gender**	*ICAM1, VCAM1, CFHR5, SERPINA5*	*ICAM1, VCAM1*
**Race**	*MSTN, RGMA, VASN, EFEMP1, PLAU, EFNA4, PLXNB2, MATN3, KITLG, CSF1, IL10RB, VCAM1, FLT3LG, ICAM1, PROC, PTPRS, PVR, NCAM1, LILRB1, CD46, CD59, SCARB2*	*ICAM1, NCAM1, VCAM1, CSF1, KITLG, HGF, PVR, SCARB2*
**MCI**	*TNFRSF12A*	
**Progressors**	*EDA2R, CCL23, ICAM1, VCAM1, SMOC2, CCL3,ACVRL1, NGF, MMP10, VASN, TNFRSF12A, MAPT, SIRT2*	*ICAM1, VCAM1, CCL3, NGF*
**CSF AD Biomarkers**		
**AB42**	*CA4, VEGFA, CHL1, ACVRL1, RGMB, EPHB6, NGF, ROBO2, IL10RB, SPARCL1, NCAM1, CD59*	*NTRK2, NCAM1, CSF1, KITLG, SCARB2*
**Tau**	*NTRK2, HGF, VASN, EPHB6, MET, RGMA, NTRK3, SOD1, TNFRSF12A, KITLG, ICAM1, CSF1, IL10RB, VCAM1, FLT3LG, PROC, CRTAC1, SCARB2, LGALS8, NCAM1, PVR, CD59, JAM2*	*ICAM1, CSF1, KITLG, NCAM1, NGF, NTRK2, SCARB2, VCAM1*
**pTau**	*NTRK2, VASN, HGF, SOD1, RGMA, NTRK3, EPHB6, MET, TNFRSF12A, KITLG, ICAM1, CSF1, IL10RB, VCAM1, FLT3LG, PROC, PTPRS, SCARB2, LGALS8, NCAM1, PVR, CD59, JAM2*	*ICAM1, CSF1, KITLG, NCAM1, NGF, NTRK2, SCARB2, VCAM1*
**TAR**	*HGF, NRP2, VASN, PLAU, LRPAP1, SOD1, NRP1, TNFRSF12A, FLT3LG, VCAM1, IL10RB, ICAM1, SCARB2*	*NCAM1, VCAM1, KITLG, HGF, NTRK2, SCARB2*

* *Abbreviations: -**MCI**: Mild Cognitive Impairment, **AD**: Alzheimer’s Disease, **Aβ42**: Amyloid-β42, **Tau**: Tau protein, **pTau**: Phosphorylated Tau protein, **TAR**: The Tau/ Aβ42 ratio, **CSF**: Cerebrospinal Fluid.
