# Supplementary material for: Cerebrospinal Fluid (CSF) Proteomic Signature in Preclinical and Clinical Alzheimer’s disease (AD): Role of Adhesion Molecules
Source: Res Sq. 2025 Jan 16:rs.3.rs-5404760. Preprint. [Version 1] doi: 10.21203/rs.3.rs-5404760/v1 (PMC11774469; doi:10.21203/rs.3.rs-5404760/v1)
Supplement: Supplement 1 [file nihpprs5404760v1-supplement-1.pdf]

Supplementary Files

This is a list of supplementary files associated with this preprint. Click to download.

- AdhesionpapersuppFinal11062024.docx
